# 2-Phenyl-1*H*-imidazol-3-ium hydrogen fumarate–fumaric acid (2/1)

**DOI:** 10.1107/S1600536812005557

**Published:** 2012-03-10

**Authors:** Hong Guo, Xin Zhang, Jian-Xia Li

**Affiliations:** a Institute of Applied Chemistry, Shenyang University of Chemical Technology, Shenyang 110142, People’s Republic of China

## Abstract

The asymmetric unit of the title compound, C_9_H_9_N_2_^+^·C_4_H_3_O_4_^−^·0.5C_4_H_4_O_4_, consists of one 2-phenyl­imidazolium cation, one hydrogen fumarate anion and half a fumaric acid mol­ecule, which lies on an inversion center. N—H⋯O and O—H⋯O hydrogen bonds connect the cations, anions and fumaric acid mol­ecules into sheets parallel to the (102) plane.

## Related literature

For similar structures, see: Jiang (2009 ▶); Song (2011 ▶); Xia & Yao (2010 ▶).
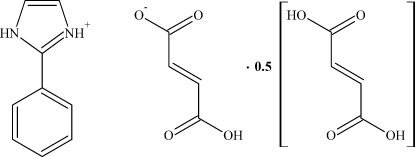


## Experimental

### 

#### Crystal data

C_9_H_9_N_2_^+^·C_4_H_3_O_4_^−^·0.5C_4_H_4_O_4_
*M* *_r_* = 318.28
Monoclinic, 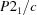

*a* = 9.572 (3) Å
*b* = 19.276 (4) Å
*c* = 8.289 (5) Å
β = 106.480 (3)°
*V* = 1466.6 (10) Å^3^
*Z* = 4
Mo *K*α radiation
μ = 0.11 mm^−1^
*T* = 293 K
0.18 × 0.15 × 0.14 mm

#### Data collection

Oxford Diffraction Gemini R Ultra diffractometer
Absorption correction: multi-scan (*CrysAlis RED*; Oxford Diffraction, 2006 ▶) *T*_min_ = 0.980, *T*_max_ = 0.984
11042 measured reflections
2675 independent reflections
1963 reflections with *I* > 2σ(*I*)

#### Refinement

*R*[*F* ^2^ > 2σ(*F* ^2^)] = 0.031
*wR*(*F* ^2^) = 0.091
*S* = 1.00
2675 reflections
209 parameters
H-atom parameters constrained
Δρ_max_ = 0.21 e Å^−3^
Δρ_min_ = −0.25 e Å^−3^

### 

Data collection: *CrysAlis CCD* (Oxford Diffraction, 2006 ▶); cell refinement: *CrysAlis CCD*; data reduction: *CrysAlis RED* (Oxford Diffraction, 2006 ▶); program(s) used to solve structure: *SHELXS97* (Sheldrick, 2008 ▶); program(s) used to refine structure: *SHELXL97* (Sheldrick, 2008 ▶); molecular graphics: *SHELXTL* (Sheldrick, 2008 ▶) and *Mercury* (Macrae *et al.*, 2006 ▶); software used to prepare material for publication: *SHELXTL*.

## Supplementary Material

Crystal structure: contains datablock(s) global, I. DOI: 10.1107/S1600536812005557/yk2037sup1.cif

Structure factors: contains datablock(s) I. DOI: 10.1107/S1600536812005557/yk2037Isup2.hkl

Supplementary material file. DOI: 10.1107/S1600536812005557/yk2037Isup3.cml

Additional supplementary materials:  crystallographic information; 3D view; checkCIF report

## Figures and Tables

**Table 1:** Hydrogen-bond geometry (Å, °)

*D*—H⋯*A*	*D*—H	H⋯*A*	*D*⋯*A*	*D*—H⋯*A*
O3—H3*A*⋯O2	0.82	1.75	2.5721 (14)	176
O5—H5*A*⋯O6^i^	0.82	1.83	2.6360 (14)	169
N1—H1*A*⋯O2	0.86	1.98	2.7903 (15)	157
N2—H2*A*⋯O1^ii^	0.86	1.86	2.7106 (14)	168

Symmetry codes: (i) 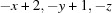; (ii) 
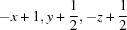
.

**Fig. 1.**
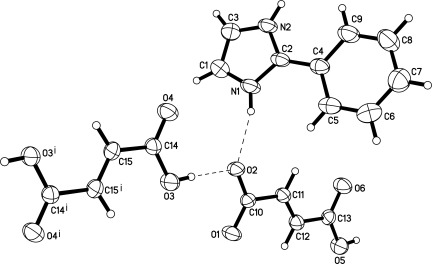
The asymmetric unit of the title compound with displacement ellipsoids drawn at the 50% probability level. Dashed lines denote hydrogen bonds. [Symmetry code: (i) -x, 1-y, 1-z.]

**Fig. 2.**
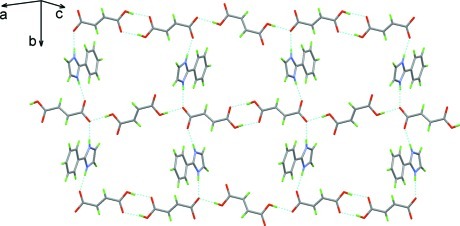
The two-dimensional network connected by hydrogen bonds (dashed lines).

## supplementary crystallographic information

### Comment

Noncovalent hydrogen-bonding interactions have attracted great interest for chemists because of their physicochemical properties (Jiang, 2009). 2-Phenylimidazole can be used to form various supramolecular architectures with anions such as acetate (Xia & Yao, 2010) and hydrogen oxalate (Song, 2011). In this work, we report the crystal structure of the title compound prepared from 2-phenylimidazole and fumaric acid.

The asymmetric unit of the title compound contains one 2-phenylimidazolium cation, one hydrogen fumarate anion and a half of fumaric acid (Fig. 1). The fumaric acid molecule lies on an inversion center. N—H···O and O—H···O hydrogen bonds connect the cations, anions and fumaric acid molecules into sheets parallel to the (1 0 2) plane (Fig. 2, Table 1).

### Experimental

2-Phenylimidazole (2 mmol), fumaric acid (1 mmol) and ethanol (15 ml) were mixed. Colorless crystals were obtained by slow evaporation of the solution at room temperature after three days.

### Refinement

All H atoms were positioned geometrically and refined as riding atoms, with C—H = 0.93, N—H = 0.86 and O—H = 0.82 Å and with *U*_iso_(H) = 1.2*U*_eq_(C, N) or 1.5*U*_eq_(O).

### Crystal data

**Table:**

C_9_H_9_N_2_^+^·C_4_H_3_O_4_^−^·0.5C_4_H_4_O_4_	*F*(000) = 664
*M**_r_* = 318.28	*D*_x_ = 1.441 Mg m^−^^3^
Monoclinic, *P*2_1_/*c*	Mo *K*α radiation, λ = 0.71073 Å
Hall symbol: -P 2ybc	Cell parameters from 2675 reflections
*a* = 9.572 (3) Å	θ = 2.2–25.3°
*b* = 19.276 (4) Å	µ = 0.11 mm^−^^1^
*c* = 8.289 (5) Å	*T* = 293 K
β = 106.480 (3)°	Block, colorless
*V* = 1466.6 (10) Å^3^	0.18 × 0.15 × 0.14 mm
*Z* = 4	

### Data collection

**Table:**

Oxford Diffraction Gemini R Ultra diffractometer	2675 independent reflections
Radiation source: fine-focus sealed tube	1963 reflections with *I* > 2σ(*I*)
Graphite monochromator	*R*_int_ = 0.000
Detector resolution: 10.0 pixels mm^-1^	θ_max_ = 25.3°, θ_min_ = 2.2°
ω scans	*h* = −11→11
Absorption correction: multi-scan (*CrysAlis RED*; Oxford Diffraction, 2006)	*k* = 0→23
*T*_min_ = 0.980, *T*_max_ = 0.984	*l* = 0→9
11042 measured reflections	

### Refinement

**Table:**

Refinement on *F*^2^	Secondary atom site location: difference Fourier map
Least-squares matrix: full	Hydrogen site location: inferred from neighbouring sites
*R*[*F*^2^ > 2σ(*F*^2^)] = 0.031	H-atom parameters constrained
*wR*(*F*^2^) = 0.091	*w* = 1/[σ^2^(*F*_o_^2^) + (0.0574*P*)^2^] where *P* = (*F*_o_^2^ + 2*F*_c_^2^)/3
*S* = 1.00	(Δ/σ)_max_ = 0.001
2675 reflections	Δρ_max_ = 0.21 e Å^−^^3^
209 parameters	Δρ_min_ = −0.25 e Å^−^^3^
0 restraints	Extinction correction: *SHELXL97* (Sheldrick, 2008), Fc^*^=kFc[1+0.001xFc^2^λ^3^/sin(2θ)]^-1/4^
Primary atom site location: structure-invariant direct methods	Extinction coefficient: 0.0068 (12)

### Special details

**Table:**

Geometry. All s.u.'s (except the s.u. in the dihedral angle between two l.s. planes) are estimated using the full covariance matrix. The cell s.u.'s are taken into account individually in the estimation of s.u.'s in distances, angles and torsion angles; correlations between s.u.'s in cell parameters are only used when they are defined by crystal symmetry. An approximate (isotropic) treatment of cell s.u.'s is used for estimating s.u.'s involving l.s. planes.
Refinement. Refinement of *F*^2^ against ALL reflections. The weighted *R*-factor *wR* and goodness of fit *S* are based on *F*^2^, conventional *R*-factors *R* are based on *F*, with *F* set to zero for negative *F*^2^. The threshold expression of *F*^2^ > σ(*F*^2^) is used only for calculating *R*-factors(gt) *etc*. and is not relevant to the choice of reflections for refinement. *R*-factors based on *F*^2^ are statistically about twice as large as those based on *F*, and *R*- factors based on ALL data will be even larger.

### Fractional atomic coordinates and isotropic or equivalent isotropic displacement parameters (Å^2^)

**Table:**

	*x*	*y*	*z*	*U*_iso_*/*U*_eq_	
C1	0.30636 (17)	0.70313 (7)	0.15392 (19)	0.0466 (4)	
H1	0.2295	0.6753	0.0948	0.056*	
C2	0.51445 (15)	0.73599 (6)	0.33290 (17)	0.0339 (3)	
C3	0.31826 (17)	0.77144 (7)	0.1375 (2)	0.0458 (4)	
H3	0.2516	0.8002	0.0642	0.055*	
C4	0.65236 (15)	0.73529 (6)	0.46476 (17)	0.0361 (3)	
C5	0.71012 (18)	0.67306 (7)	0.53922 (19)	0.0441 (4)	
H5	0.6609	0.6317	0.5041	0.053*	
C6	0.83936 (19)	0.67245 (8)	0.6642 (2)	0.0531 (4)	
H6	0.8772	0.6306	0.7138	0.064*	
C7	0.91375 (19)	0.73335 (8)	0.7170 (2)	0.0559 (4)	
H7	1.0020	0.7325	0.8011	0.067*	
C8	0.85745 (19)	0.79517 (8)	0.6455 (2)	0.0554 (4)	
H8	0.9075	0.8363	0.6817	0.067*	
C9	0.72735 (18)	0.79677 (7)	0.52033 (19)	0.0464 (4)	
H9	0.6894	0.8389	0.4728	0.056*	
C10	0.50312 (14)	0.48691 (6)	0.25178 (15)	0.0284 (3)	
C11	0.63137 (14)	0.50545 (6)	0.19048 (15)	0.0316 (3)	
H11	0.6584	0.5519	0.1930	0.038*	
C12	0.70748 (14)	0.46016 (7)	0.13372 (15)	0.0329 (3)	
H12	0.6801	0.4138	0.1308	0.039*	
C13	0.83360 (14)	0.47805 (7)	0.07425 (15)	0.0299 (3)	
C14	0.13846 (14)	0.54984 (6)	0.41335 (15)	0.0306 (3)	
C15	0.01299 (14)	0.53111 (7)	0.47557 (15)	0.0325 (3)	
H15	−0.0525	0.5661	0.4804	0.039*	
N1	0.42847 (13)	0.68174 (5)	0.27399 (15)	0.0414 (3)	
H1A	0.4472	0.6396	0.3068	0.050*	
N2	0.44703 (13)	0.79138 (5)	0.24890 (14)	0.0389 (3)	
H2A	0.4796	0.8332	0.2627	0.047*	
O1	0.46797 (11)	0.42536 (4)	0.25506 (12)	0.0448 (3)	
O2	0.43619 (9)	0.53724 (4)	0.29443 (11)	0.0356 (2)	
O3	0.21778 (10)	0.49744 (5)	0.39383 (12)	0.0462 (3)	
H3A	0.2850	0.5114	0.3593	0.055*	
O4	0.16258 (11)	0.60952 (5)	0.38435 (13)	0.0482 (3)	
O5	0.90474 (11)	0.42773 (5)	0.03852 (12)	0.0447 (3)	
H5A	0.9724	0.4427	0.0065	0.054*	
O6	0.86586 (11)	0.54051 (5)	0.06280 (12)	0.0453 (3)	

### Atomic displacement parameters (Å^2^)

**Table:**

	*U*^11^	*U*^22^	*U*^33^	*U*^12^	*U*^13^	*U*^23^
C1	0.0451 (10)	0.0360 (8)	0.0582 (9)	−0.0057 (7)	0.0139 (8)	−0.0014 (7)
C2	0.0424 (9)	0.0209 (6)	0.0464 (8)	−0.0019 (6)	0.0256 (7)	0.0009 (6)
C3	0.0454 (10)	0.0354 (8)	0.0562 (9)	0.0006 (6)	0.0137 (8)	0.0061 (7)
C4	0.0423 (9)	0.0277 (7)	0.0453 (8)	−0.0013 (6)	0.0241 (7)	0.0002 (6)
C5	0.0561 (11)	0.0288 (7)	0.0516 (9)	0.0015 (6)	0.0221 (8)	0.0008 (6)
C6	0.0614 (12)	0.0431 (9)	0.0549 (10)	0.0113 (8)	0.0165 (9)	0.0079 (7)
C7	0.0523 (11)	0.0583 (11)	0.0544 (10)	0.0015 (8)	0.0106 (8)	0.0029 (8)
C8	0.0592 (12)	0.0439 (9)	0.0607 (10)	−0.0144 (8)	0.0131 (9)	−0.0017 (8)
C9	0.0550 (11)	0.0289 (8)	0.0565 (9)	−0.0039 (7)	0.0176 (8)	0.0036 (7)
C10	0.0279 (7)	0.0247 (7)	0.0349 (7)	0.0016 (5)	0.0125 (6)	0.0001 (5)
C11	0.0305 (8)	0.0263 (6)	0.0411 (8)	−0.0017 (5)	0.0150 (6)	−0.0001 (5)
C12	0.0300 (8)	0.0292 (7)	0.0428 (8)	−0.0025 (5)	0.0156 (6)	−0.0018 (6)
C13	0.0266 (7)	0.0314 (7)	0.0332 (7)	0.0007 (6)	0.0109 (6)	−0.0024 (5)
C14	0.0297 (7)	0.0290 (7)	0.0351 (7)	−0.0016 (6)	0.0122 (6)	−0.0021 (5)
C15	0.0279 (7)	0.0335 (6)	0.0394 (7)	0.0011 (6)	0.0150 (6)	−0.0023 (6)
N1	0.0488 (8)	0.0220 (6)	0.0566 (8)	−0.0036 (5)	0.0202 (6)	0.0024 (5)
N2	0.0456 (8)	0.0211 (5)	0.0543 (7)	−0.0024 (5)	0.0211 (6)	0.0033 (5)
O1	0.0463 (6)	0.0222 (5)	0.0781 (7)	−0.0021 (4)	0.0375 (6)	−0.0028 (4)
O2	0.0352 (6)	0.0235 (5)	0.0566 (6)	0.0021 (4)	0.0271 (5)	−0.0006 (4)
O3	0.0433 (6)	0.0322 (5)	0.0782 (7)	0.0002 (4)	0.0418 (6)	0.0038 (5)
O4	0.0498 (7)	0.0303 (6)	0.0741 (7)	−0.0033 (4)	0.0329 (6)	0.0027 (5)
O5	0.0381 (6)	0.0384 (6)	0.0694 (7)	0.0005 (4)	0.0343 (5)	−0.0041 (5)
O6	0.0446 (6)	0.0320 (6)	0.0692 (7)	−0.0033 (4)	0.0324 (5)	−0.0017 (5)

### Geometric parameters (Å, º)

**Table:**

C1—C3	1.332 (2)	C10—O1	1.2357 (14)
C1—N1	1.3663 (19)	C10—O2	1.2669 (15)
C1—H1	0.9300	C10—C11	1.4989 (18)
C2—N1	1.3346 (16)	C11—C12	1.3072 (18)
C2—N2	1.3366 (17)	C11—H11	0.9300
C2—C4	1.456 (2)	C12—C13	1.4686 (18)
C3—N2	1.3687 (19)	C12—H12	0.9300
C3—H3	0.9300	C13—O6	1.2532 (15)
C4—C5	1.3899 (19)	C13—O5	1.2676 (15)
C4—C9	1.3944 (19)	C14—O4	1.2108 (15)
C5—C6	1.370 (2)	C14—O3	1.3009 (15)
C5—H5	0.9300	C14—C15	1.4799 (18)
C6—C7	1.378 (2)	C15—C15^i^	1.312 (3)
C6—H6	0.9300	C15—H15	0.9300
C7—C8	1.372 (2)	N1—H1A	0.8592
C7—H7	0.9300	N2—H2A	0.8602
C8—C9	1.377 (2)	O3—H3A	0.8203
C8—H8	0.9300	O5—H5A	0.8193
C9—H9	0.9300		
			
C3—C1—N1	107.00 (13)	O1—C10—O2	124.52 (12)
C3—C1—H1	126.5	O1—C10—C11	119.39 (11)
N1—C1—H1	126.5	O2—C10—C11	116.08 (11)
N1—C2—N2	106.19 (12)	C12—C11—C10	123.81 (12)
N1—C2—C4	126.96 (11)	C12—C11—H11	118.1
N2—C2—C4	126.84 (11)	C10—C11—H11	118.1
C1—C3—N2	107.20 (13)	C11—C12—C13	124.00 (12)
C1—C3—H3	126.4	C11—C12—H12	118.0
N2—C3—H3	126.4	C13—C12—H12	118.0
C5—C4—C9	118.96 (14)	O6—C13—O5	123.87 (12)
C5—C4—C2	120.21 (12)	O6—C13—C12	119.64 (11)
C9—C4—C2	120.82 (12)	O5—C13—C12	116.49 (11)
C6—C5—C4	120.22 (13)	O4—C14—O3	124.20 (12)
C6—C5—H5	119.9	O4—C14—C15	121.35 (12)
C4—C5—H5	119.9	O3—C14—C15	114.45 (11)
C5—C6—C7	120.47 (14)	C15^i^—C15—C14	125.05 (16)
C5—C6—H6	119.8	C15^i^—C15—H15	117.5
C7—C6—H6	119.8	C14—C15—H15	117.5
C8—C7—C6	119.90 (16)	C2—N1—C1	109.96 (11)
C8—C7—H7	120.0	C2—N1—H1A	125.0
C6—C7—H7	120.0	C1—N1—H1A	125.1
C7—C8—C9	120.39 (14)	C2—N2—C3	109.65 (11)
C7—C8—H8	119.8	C2—N2—H2A	125.2
C9—C8—H8	119.8	C3—N2—H2A	125.2
C8—C9—C4	120.05 (13)	C14—O3—H3A	109.4
C8—C9—H9	120.0	C13—O5—H5A	109.5
C4—C9—H9	120.0		
			
N1—C1—C3—N2	−0.55 (17)	O1—C10—C11—C12	2.4 (2)
N1—C2—C4—C5	2.4 (2)	O2—C10—C11—C12	−176.38 (12)
N2—C2—C4—C5	−179.07 (12)	C10—C11—C12—C13	−179.71 (11)
N1—C2—C4—C9	−176.51 (13)	C11—C12—C13—O6	−6.1 (2)
N2—C2—C4—C9	2.0 (2)	C11—C12—C13—O5	173.31 (12)
C9—C4—C5—C6	−0.5 (2)	O4—C14—C15—C15^i^	−173.42 (16)
C2—C4—C5—C6	−179.49 (13)	O3—C14—C15—C15^i^	6.5 (2)
C4—C5—C6—C7	−0.3 (2)	N2—C2—N1—C1	−0.52 (15)
C5—C6—C7—C8	0.7 (2)	C4—C2—N1—C1	178.24 (13)
C6—C7—C8—C9	−0.4 (3)	C3—C1—N1—C2	0.67 (17)
C7—C8—C9—C4	−0.4 (2)	N1—C2—N2—C3	0.17 (15)
C5—C4—C9—C8	0.9 (2)	C4—C2—N2—C3	−178.59 (12)
C2—C4—C9—C8	179.82 (13)	C1—C3—N2—C2	0.24 (16)

Symmetry code: (i) −*x*, −*y*+1, −*z*+1.

### Hydrogen-bond geometry (Å, º)

**Table:**

*D*—H···*A*	*D*—H	H···*A*	*D*···*A*	*D*—H···*A*
O3—H3*A*···O2	0.82	1.75	2.5721 (14)	176
O5—H5*A*···O6^ii^	0.82	1.83	2.6360 (14)	169
N1—H1*A*···O2	0.86	1.98	2.7903 (15)	157
N2—H2*A*···O1^iii^	0.86	1.86	2.7106 (14)	168

Symmetry codes: (ii) −*x*+2, −*y*+1, −*z*; (iii) −*x*+1, *y*+1/2, −*z*+1/2.
